# The Association between the Preference for Active Play and Neurological Development in Toddlers: A Register-Based Study

**DOI:** 10.3390/ijerph17072525

**Published:** 2020-04-07

**Authors:** Anni Pakarinen, Lea Hautala, Lotta Hamari, Minna Aromaa, Hannele Kallio, Pirjo-Riitta Liuksila, Matti Sillanpää, Sanna Salanterä

**Affiliations:** 1 Department of Nursing Science, University of Turku, 20014 Turku, Finland; 2 Department of Clinical Medicine, University of Turku, 20014 Turku, Finland; 3 Department of Public Health, University of Turku, 20014 Turku, Finland; 4 Health care services, Welfare Division, City of Turku, PO Box 670, 20101 Turku, Finland; 5 Family and social services, Welfare Division, City of Turku, PO Box 670, 20101 Turku, Finland; 6 Departments of General Practice and Child Neurology, University of Turku, 20014 Turku, Finland; 7 Turku University Hospital, PO Box 52, 20521 Turku, Finland

**Keywords:** physical activity, active play, early childhood, toddlerhood, gross motor competence, neurological development

## Abstract

Active play is regarded as physical activity during early childhood. Physical activity has many benefits for children’s physical and psychosocial health and wellbeing, as well as for their cognitive development. The aim of this study was to investigate associations between the preference for active play and neurological development in toddlers. The study was conducted as a register-based study, and the data were collected from a public-health clinic’s electronic health records. The register data about active play used in this study were originally assessed by parents at home and by early years teachers at nurseries. Neurological development was assessed by the public health nurses in public child-health clinics. The data eligible for this study were available from 717 toddlers aged 2.5–3.0 years old (mean: 2.5 years ± 2 months). The majority of toddlers (85%) showed a preference for active play, both at home and at the nursery. The prevalence of delays in the neurological development of toddlers varied in different developmental areas (by 1–15%). Delays in gross motor competence, auditory perception, and self-help skills were associated with a lower preference for active play in nursery settings, but none of the neurodevelopmental items were found to be associated with toddlers’ preference for active play at home. Nurseries need to encourage children to actively play and support their gross motor competence and self-help skills.

## 1. Introduction

A physically active way of life develops early on in childhood [1,2]. Physical activity has many benefits for children’s overall health [3], psychosocial wellbeing [4], and cognitive development [5]. However, most small children are not physically active enough to gain these benefits [6,7].

Active play, defined as physical activity in a playful context [8], is regarded as physical activity during early childhood (since it generally forms most of children’s daily physical activity) [9]. “Active play” usually refers to free-flow play, following the child’s preferences and intrinsic motivations, and it is distinguished from more structured physical activity [10]. Active play challenges children’s creativity and involves improvised forms of activity. It can be influenced on multiple levels by society and environments. Active play is possible almost anywhere, regardless of the availability of preset plans. In addition, active play is often engaged in outdoors, and the time spent outdoors has been shown to be related to the amount of physical activity of small children [11,12]. Encouraging and offering children possibilities to participate in active play may have an important contribution to children’s overall physical activity levels [13,14,15,16]. Offering different types of surroundings is important, since the amount of active play of moderate-to-vigorous intensity varies depending on the environment [16].

Earlier studies have indicated that school-aged children (6–12 years old) with a developmental coordination disorder [17,18,19] and other disabilities—such as physical, intellectual, or sensory disabilities [20,21]—participate in less physical activity compared to their typically developing peers. Little evidence is available on these associations during early childhood. Only a few studies have examined these associations, and according to them, gross motor impairments are associated with lower levels of participation in physical activity among three- to four-year-old children [22,23]. Both studies measured physical activity using accelerometers.

There is lack of knowledge of the associations between neurological development and the preference for active play among toddlers (1–3 years old). Considering the rapid changes in neurodevelopment at the toddler age, we wanted to limit the age range to a shorter period of 2.5–3.0 years old, and thus minimise the drawbacks of inter-subject developmental variety in the analyses.

The aim of this study was to investigate the associations between the preference for active play and neurological development in toddlers. Furthermore, the aim is to describe the inter-observer variation between parents’ and early years teachers’ assessments. Our hypothesis was that delays in neurological development are associated with lower preference for active play, as assessed by both the parents and early years teachers.

## 2. Materials and Methods

The study was conducted as a register-based study. The register data were collected from a public-health clinic’s electronic health records. The study follows the methodological guidelines for cohort studies outlined in the “Strengthening the Reporting of Observational Studies in Epidemiology” (STROBE) statement [24].

### 2.1. The Sample and Setting

**Figure 1:**
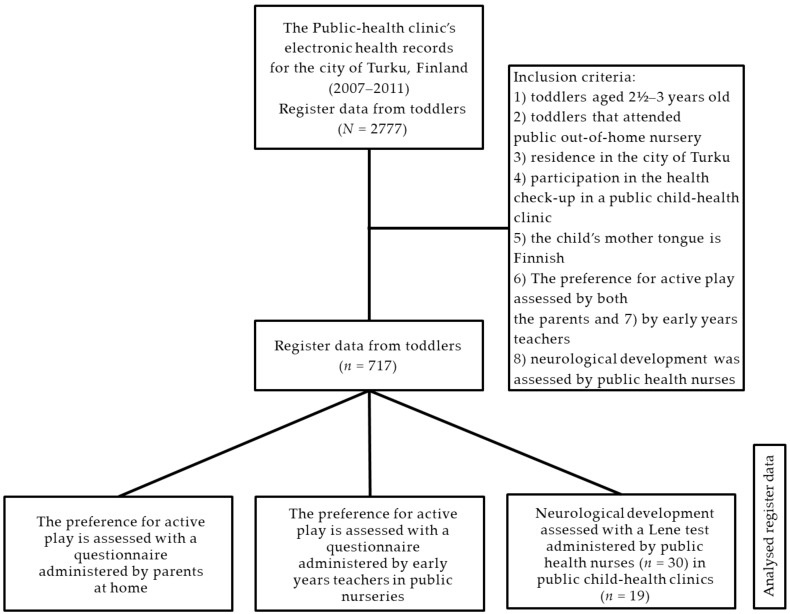
A flow chart of the research design.

The register consisted of data on toddlers aged 2.5–3.0 years old that regularly attended a public, out-of-home nursery (later referred to as “nursery”) (*n* = 2777) during the years 2007–2011 in the city of Turku. The inclusion criteria were: (1) toddlers aged 2.5–3.0 years old; (2) toddlers that attended a public, out-of-home nursery; (3) the residence of the child’s family in the city of Turku; (4) participation in the regular 2.5-year-old health check-up at a public child-health clinic; (5) the child’s mother tongue is Finnish; (6) the child’s preference for active play was assessed by parents at home; (7) the child’s preference for active play was assessed by early years teachers in a public, out-of-home nurseries; and (8) neurological development was assessed by public health nurses in public child-health clinics in the city of Turku. A total of 717 children (*n* = 717; girls: *n* = 349, 49%; boys: *n* = 368, 51%) met the criteria and were included in the study (mean age: 2.5 years old; standard deviation (SD): 0.2 years) (see Figure 1).

### 2.2. Data Collection

The child’s preference for active play was originally assessed with questionnaires administered by parents and the early years teachers. The preference for active play was inquired about by using the following statement: “the child participates willingly in active play”. Assessors were instructed to choose the response option that best described the child’s current behaviour in relation to the statement. The items were scored as follows: “most of the time” (score: 0), “variably” (score: 1), or “not yet” (score: 2). The questionnaire data were saved to the electronic health records in the public health clinic during routine appointments and collected from there for this study.

Children’s neurological development was originally assessed by the public health nurses (*n* = 30) in public child-health clinics (*n* = 19) by applying the Lene test, which is a neurodevelopmental screening tool for toddlers and preschoolers. The Lene test covers all of the essential areas of neurological development: the attention–behaviour area, the motor–perceptual area, and language. The validity (structural, concurrent, and predictive validity) and reliability (test–retest reliability and internal consistency) of the test have been studied in previous studies, and the scores were within acceptable limits [22,23,24]. The Lene test consists of 12 items, five of which consist of tasks for the child to execute. The items are the following: visual perception (four tasks), hearing, gross motor competence (five tasks), interactional skills, attention and motivation, expressive speech, understanding speech and concepts (four tasks), auditory perception (four tasks), eye–hand co-ordination (three tasks), play skills, and self-help skills. For example, the gross motor competence tasks include walking, toe walking, standing on one leg, jumping, and throwing a ball; eye–hand co-ordination tasks include building a block tower, reproducing a drawing, and unscrewing a cap. The items were scored on scale of 0–3. Zero (0) indicates normal development, one (1) indicates suspect or mild delay, two (2) indicates moderate or severe delay, and three (3) indicates refusal to complete the task. The Lene test takes about 30 min to administer. Public health nurses were trained to administer the Lene test by the Lene test developer [25,26,27]. The data collected with the Lene test were saved to the electronic health records in the public health clinic during routine appointments and collected from there for this study.

### 2.3. Statistical Analysis

In the preliminary analyses, children with an assessed preference for active play were compared with children who were not assessed using crosstabulations and the χ2-test or Fisher’s exact test. No significant differences were found between the 717 subjects and 1554 non-subjects regarding sex and neurological development (all *p* values ≥ 0.01). Descriptive statistics were used to estimate the prevalence of the delays in neurological development and in the preference for active play. Crosstabulations and Fisher’s exact tests were applied to investigate the associations between the explanatory variables (visual perception, hearing, gross motor competence, interactional skills, attention and motivation, expressive speech, understanding speech and concepts, auditory perception, eye–hand coordination, play skills, and self-help skills) and the response variables (the preference for active play at home and at nursery). Due to the small number of children with moderate or severe delays in neurological development, the classes of “suspect or mild delay” and “moderate or severe delay” were combined for the analyses.

The difference between the parents’ and early years teachers’ assessments was estimated by computing the proportion of agreement (P), as presented by Grant [28]. For the analyses, response options indicating variability in the child’s willingness to play or a lack of willingness to play were combined, due to the small number of responses in the latter class. Deeper insight into disagreements between the parents and early years teachers were obtained by testing which of the explanatory variables associated with the child’s preference for active play were associated with the disagreement. The analyses were first conducted by using Fisher’s exact tests to find factors associated with the disagreement, and then by using multivariable logistic regression analysis to identify the factors that were independently associated with the disagreement. In the multivariable model, the explanatory variables consisted of those variables that were significant in the first analyses. The statistical computations were performed with the SAS system for Windows, release 9.3. Due to the explorative nature of the study, *p* values < 0.01 were interpreted as being significant.

### 2.4. Ethical Considerations

The study design was approved by the Institutional Review Board of the city of Turku. The study subjects or relatives of the study subjects were not personally contacted for the present study. According to Finnish legislation [29], no approval by an ethical committee or informed consent from the study subjects are required for studies based purely on data files that are statistical and provided for scientific research purposes without identification information.

## 3. Results

The prevalence of delays in the neurological development of children varied in different developmental areas from less than 1% to more than 15%. More than 10% of the children showed delayed eye–hand co-ordination, attention and motivation, expressive speech, interactional skills, and self-help skills (Table 1).

Altogether, 15% (108/717) of the children showed variability in their preference for active play or a lack of a preference to actively play: for 3% (23/717), this was only at home; for 9% (64/717), this was only at nursery; and for another 3% (21/717), this was the case both at home and at nursery. At nursery, the preference for active play was less common in children with a delay in gross motor competence (*p* = 0.005), auditory perception (*p* = 0.006), and self-help skills (*p* = 0.002) than in those with normal development (see Table 2). None of these items were associated with the children’s preference for active play at home (see Table 3).

For 12% (87/717) of the children, parents’ assessments concerning the children’s preference for active play differed from those of the early years teachers (*p* < 0.001). At nursery, the variability or lack of preference for active play was twice as common (12%, 85/717) than at home (6%, 44/717). In 88% (609/696) of the responses that indicated that the child showed a preference for active play most of the time, parents and early years teachers gave similar assessments (P = 0.875; 95% CI 0.848–0.899), whereas lower agreement (19%, 21/108) was found in responses indicating the variability of the preference for active play or a lack of preference for active play (P = 0.194; 95% CI: 0.125–0.282).

Neurodevelopmental items that were associated with disagreements in the assessments of the children’s preference for active play between those of the early years teachers and those of the parents were children’s gross motor competence (*p* = 0.005), auditory perception (*p* < 0.001) and self-help skills (*p* < 0.001). In multivariable analysis, self-help skills had an independent association with disagreement concerning children’s preference for active play (*OR*: 3.1; 99% CI: 1.3–7.4; *p* = 0.001), while gross motor competence and auditory perception were not significant (*p* values ≥ 0.010). Children with a delay in self-help skills had two times higher odds of being classified into a different group regarding their preference for active play by the early years teachers compared with the parents. For those children, the variability or lack of preference for active play was relatively more common at nursery (24%; 18/74) than at home (5%; 4/74).

## 4. Discussion

This study investigated the associations between a preference for active play and neurological development among toddlers based on register data. We also investigated the inter-observer variation between parents’ and early years teachers’ assessments. The results suggest that the majority of toddlers were willing to participate in active play at home, and that delays in neurological development were not associated with a preference for active play, as assessed by the parents. At nurseries, their participation was reported to be less common, and delays in gross motor competence, auditory perception, and self-help skills were associated with a lower preference for active play, as assessed by the early years teachers. The variation between the parents’ and early years teachers’ assessments was significant, and differed most strongly among toddlers with delayed self-help skills. These results might be due to differences between the parents and teachers in their understanding of “participates willingly in active play”. Understandably, the parents are at higher risk of overestimating their children’s performances than the teachers. On the other hand, children may actually be more active at home because of the possible encouragement and support of their parents, factors which are positively associated with children’s physical activity behaviour [5,30,31,32,33,34]. Furthermore, home and nursery settings may offer very different opportunities and environments for active play—such as outdoor activities and playgrounds, free-flow play, and play equipment—which are associated with children’s physical activity [11,12,13,16,33,35,36,37,38,39,40].

The majority of previous studies have concentrated on school-aged children’s delays or disabilities in neurological development, and the associations with their participation in physical activity [17,18,19,20,21,30]. Our findings are partly in accordance with earlier studies among three- to four-year-old preschoolers [22,23], confirming that delayed gross motor competence is shown to already be associated with lower levels of active play during toddlerhood; however, the results are only consistent with the early years teacher’s assessments. Children’s neurodevelopmental delays are usually continuous or persistent and may predict learning disabilities at school [26,41,42]. Detecting and addressing these delays in their early phase helps health professionals to make decisions about supportive and need-based interventions for children well before school starts [43,44]. While investing in evidence-based interventions, new approaches should also be explored. The present study took a novel approach and investigated whether active play had associations with neurological development in order to enable early intervention in case of delay.

The present study has some limitations. First, a relatively small proportion (26%) of the available data met the inclusion criteria, since only data with both the parents’ and early years teachers’ assessments could be included. However, the non-subjects did not significantly differ from the study subjects. Second, the subjective assessment of the preference for active play is considered a limitation. However, earlier studies indicate that parents, especially mothers [1] and early years teachers [45], can be considered as capable and valid sources in assessing small children’s physical activity behaviour.

## 5. Conclusions

Follow-up on and the promotion of children’s neurological development, as well as the prediction and detection of delays, are integral parts of childcare in families and public child health units. A preference for active play at the toddler age seems to be an indicator of later neurological development in the nursery setting. Active play, measured by the nursery professionals, significantly predicts later overall gross motor development, self-help skills, and auditory perception. This observation emphasises the importance of the motor activities of a developing child in our era, when children are increasingly fond of sedentary hobbies and are exposed to their adverse health effects. Attention should be paid to having sufficient facilities for active play and exercise games for children in nursery settings and other settings.

## Figures and Tables

**Table 1:** The distribution of neurological variables in 2.5-year-old children (*n* = 717), as measured by the Lene test.

Variable	Neurodevelopmental Status	
	Normal (%)	Delayed (%)
Hearing (*n* = 668)	99.6	0.4
Visual perception (*n* = 614)	98.4	1.6
Gross motor competence (*n* = 647)	96.1	3.9
Understanding speech and concepts (*n* = 668)	95.4	4.6
Play skills (*n* = 698)	94.7	5.3
Auditory perception (*n* = 660)	93.8	6.2
Self-help skills (*n* = 692)	89.3	10.7
Interactional skills (*n* = 709)	89.0	11.0
Expressive speech (*n* = 699)	88.1	11.9
Attention and motivation (*n* = 708)	84.6	15.4
Eye–hand coordination (*n* = 660)	84.2	15.8

**Table 2:** The association of the neurological developmental variables with the preference for active play in 2.5-year-old children (*n* = 717) in a nursery.

Variable	Preference for Active Play		
	Present Most of the Time (%)	Varies/Not Yet (%)	*p-*Value ^a^
Hearing			0.311
Abnormal (*n* = 3)	67	33	
Normal (*n* = 665)	88	12	
Visual perception			1.000
Abnormal (*n* = 10)	90	10	
Normal (*n* = 604)	86	14	
Gross motor competence			**0.005**
Delayed (*n* = 25)	68	32	
Normal (*n* = 622)	98	11	
Understanding speech and concepts			0.086
Delayed (*n* = 31)	77	23	
Normal (*n* = 637)	88	12	
Play skills			0.189
Delayed (*n* = 37)	81	19	
Normal (*n* = 661)	89	11	
Auditory perception			**0.006**
Delayed (*n* = 41)	73	27	
Normal (*n* = 619)	89	11	
Self-help skills			**0.002**
Delayed (*n* = 74)	76	24	
Normal (*n* = 618)	89	11	
Interactional skills			0.043
Delayed (*n* = 78)	81	19	
Normal (*n* = 631)	89	11	
Expressive speech			0.147
Delayed (*n* = 83)	83	17	
Normal (*n* = 616)	89	11	
Attention and motivation			0.076
Delayed (*n* = 109)	83	17	
Normal (*n* = 599)	89	11	
Eye–hand coordination			0.050
Delayed (*n* = 104)	82	18	
Normal (*n* = 556)	89	11	

^a^ Fisher’s exact test. The statistically significant values are marked in bold.

**Table 3:** The association of the neurological developmental variables with the preference for active play of 2.5-year-old children (*n* = 717) at home.

Variable	Preference to Active Play		
	Most of the Time (%)	Varies/not yet (%)	*p*-Value ^a^
Hearing			0.177
Abnormal (*n* = 3)	67	33	
Normal (*n* = 665)	94	6	
Visual perception			1.000
Abnormal (*n* = 10)	100	0	
Normal (*n* = 604)	94	6	
Gross motor competence			0.044
Delayed (*n* = 25)	84	16	
Normal (*n* = 622)	95	5	
Understanding speech and concepts			1.000
Delayed (*n* = 31)	97	3	
Normal (*n* = 637)	94	6	
Play skills			0.276
Delayed (*n* = 37)	89	11	
Normal (*n* = 661)	94	6	
Auditory perception			0.022
Delayed (*n* = 41)	85	15	
Normal (*n* = 619)	95	5	
Self-help skills			1.000
Delayed (*n* = 74)	95	5	
Normal (*n* = 618)	94	6	
Interactional skills			0.315
Delayed (*n* = 78)	91	9	
Normal (*n* = 631)	94	6	
Expressive speech			0.213
Delayed (*n* = 83)	98	2	
Normal (*n* = 616)	94	6	
Attention and motivation			0.665
Delayed (*n* = 109)	93	7	
Normal (*n* = 599)	94	6	
Eye-hand co-ordination			0.646
Delayed (*n* = 104)	93	7	
Normal (*n* = 556)	94	6	

^a^ Fisher’s exact test.
